# Consensus Micro RNAs Governing the Switch of Dormant Tumors to the Fast-Growing Angiogenic Phenotype

**DOI:** 10.1371/journal.pone.0044001

**Published:** 2012-08-31

**Authors:** Nava Almog, Lili Ma, Christian Schwager, Bastian G. Brinkmann, Afshin Beheshti, Peter Vajkoczy, Judah Folkman, Lynn Hlatky, Amir Abdollahi

**Affiliations:** 1 Center of Cancer Systems Biology, Steward Research & Specialty Projects Corp., St. Elizabeth's Medical Center, Tufts University School of Medicine, Boston, Massachusetts, United States of America; 2 Molecular and Translational Radiation Oncology, Heidelberg Ion Therapy Center, University of Heidelberg Medical School and National Center for Tumor diseases, German Cancer Research Center, Heidelberg, Germany; 3 Department of Neurosurgery, Charité – Universitaetsmedizin Berlin, Berlin, Germany; 4 Department of Surgery, Harvard Medical School and Vascular Biology Program, Children's Hospital, Boston, Massachusetts, United States of America; Ospedale Pediatrico Bambino Gesù, Italy

## Abstract

Tumor dormancy refers to a critical stage in cancer development in which tumor cells remain occult for a prolonged period of time until they eventually progress and become clinically apparent. We previously showed that the switch of dormant tumors to fast-growth is angiogenesis dependent and requires a stable transcriptional reprogramming in tumor cells. Considering microRNAs (miRs) as master regulators of transcriptome, we sought to investigate their role in the control of tumor dormancy. We report here the identification of a consensus set of 19 miRs that govern the phenotypic switch of human dormant breast carcinoma, glioblastoma, osteosarcoma, and liposarcoma tumors to fast-growth. Loss of expression of dormancy-associated miRs (DmiRs, 16/19) was the prevailing regulation pattern correlating with the switch of dormant tumors to fast-growth. The expression pattern of two DmiRs (miR-580 and 190) was confirmed to correlate with disease stage in human glioma specimens. Reconstitution of a single DmiR (miR-580, 588 or 190) led to phenotypic reversal of fast-growing angiogenic tumors towards prolonged tumor dormancy. Of note, 60% of angiogenic glioblastoma and 100% of angiogenic osteosarcoma over-expressing miR190 remained dormant during the entire observation period of ∼ 120 days. Next, the ability of DmiRs to regulate angiogenesis and dormancy-associated genes was evaluated. Transcriptional reprogramming of tumors via DmiR-580, 588 or 190 over-expression resulted in downregulation of pro-angiogenic factors such as TIMP-3, bFGF and TGFalpha. In addition, a G-CSF independent downregulation of Bv8 was found as a common target of all three DmiRs and correlated with decreased tumor recruitment of bone marrow-derived CD11b+ Gr-1+ myeloid cells. In contrast, antiangiogenic and dormancy promoting pathways such as EphA5 and Angiomotin were upregulated in DmiR over-expressing tumors. This work suggests novel means to reverse the malignant tumor phenotype into an asymptomatic dormant state and may provide promising targets for early detection or prevention of cancer.

## Introduction

Tumor dormancy is an early stage in cancer progression in which small cancerous lesions (few millimeters in diameter) remain occult and asymptomatic until they eventually switch to become fast-growing, clinically apparent and potentially lethal cancer. Dormant cancerous lesions are highly prevalent in asymptomatic normal populations [1]. However, the majority of these lesions never progress to the stage of exponential tumor growth. This implies that dormant tumors rarely succeed in overcoming inherent defense mechanisms against tumor development. These include cell cycle arrest of disseminated tumor cells (DTCs), tumor cell senescence, immune response of the host, hormonal control or the insufficiency of dormant tumors to recruit new blood vessels [2]–[14].

Of particular clinical importance is the fact that dormant tumor cells left after primary tumor removal or treatment may contribute to tumor relapse and are often refractory to cancer therapies [2]. Although the tumor dormancy phase is a promising therapeutic target, it is still one of the most neglected areas in cancer biology. This is mainly due to lack of suitable experimental models and limited clinical accessibility to dormant tumors.

We have successfully established *in-vivo* models of vascular tumor dormancy of human breast cancer, glioblastoma, osteosarcoma, and liposarcoma in immunocompromised mice [15], [16]. In these models, tumor dormancy is characterized by high proliferation of tumor cells balanced by apoptosis and impaired tumor angiogenesis. Tumors remain dormant for a prolonged period of time until they spontaneously switch to the fast-growing angiogenic phenotype. These angiogenic tumors retain their ability to grow fast once injected in new mice. Therefore, we hypothesized that dormant tumors undergo a stable genetic “reprogramming” during their transition from dormancy to fast-growth [17]. Although the mechanisms triggering the transition from dormancy to rapid growth remain to be elucidated, pathways promoting tumor dormancy and the transcriptional switch of dormant tumors to the fast-growing phenotype was recently deciphered by genome-wide expression analysis [17].

MicroRNAs are considered potential master regulators” of gene expression [18], [19]. In a hierarchical manner, a single microRNA (miR) could regulate the expression level of multiple target genes. Therefore, we sought to characterize the ‘master regulators’ of tumor dormancy by utilizing the *in-vivo* models established in our lab.

**Figure 1:**
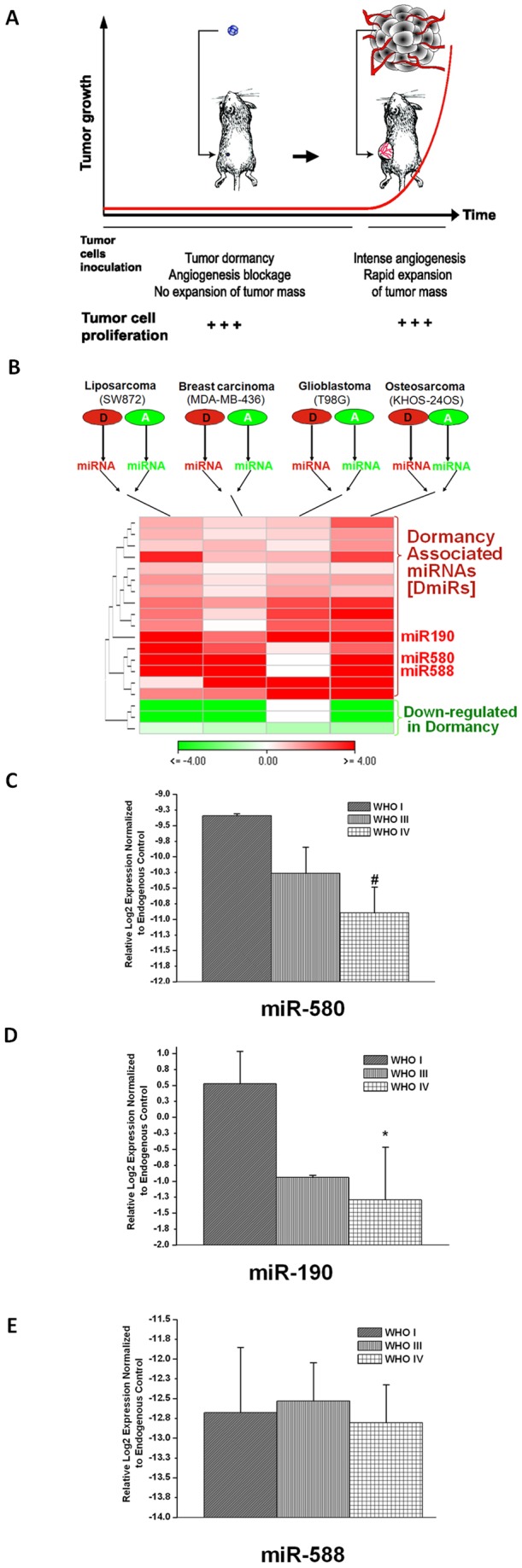
Consensus signature of dormancy associated miRs. A schematic overview of the *in-vivo* experimental vascular tumor dormancy models employed (A). Dormant tumors are formed after s.c. injection of tumor cells in immunocompromised mice and remain undetectable by gross examination for a prolonged period of time. This dormancy period is characterized by impaired tumor angiogenesis and high levels of cell turnover, i.e., balanced tumor cell proliferation and apoptosis. Tumors spontaneously exit the dormancy phase and switch to a rapid “angiogenic” growth. Differential regulation of miRs in dormant vs. fast-growing tumors (B). 19 miRs were significantly differentially regulated during the switch of dormant tumors to fast-growth in all four dormancy models (p<0.03). The prevailing regulation pattern was the loss of dormancy associated miRs (DmiRs) after the switch of dormant tumors. The heatmap represents fold expression values of miRs in dormant vs. fast-growing tumors according to the scale bar. Red: miRs upregulated in dormant tumors. Green: miRs down-regulated in dormant tumors. MiRs are sorted by hierarchical clustering. The expression level of miR-580, miR-588 and miR-190 was detected in human glioma specimens (C–E). In support of the experimental data, the expression of miR-580 and miR-190 was significantly decreased with advanced tumor grade in glioma specimens. WHO-I (n:3), WHO-III (n:4) and WHO-IV (n:8). *p<0.01, #p = 0.05.

Here we report the identification of a consensus miR signature that governs tumor dormancy. A concerted shift in expression of these miRs correlated with the conversion of all four dormant tumors to the fast-growing angiogenic phenotype. Expression patterns of candidate dormancy miRs in tumor cells were correlated with disease stage in glioma patients. We further show that reconstitution of single candidate tumor dormancy-associated miRs (DmiRs) in fast-growing glioblastoma or osteosarcoma cells resulted in significant inhibition of tumor growth. Over-expression of the three DmiRs also led to suppression of pro-angiogenic and upregulation of antiangiogenic genes and correlated with marked inhibition of tumor growth and prolonged dormancy periods. Moreover, the expression of DmiRs led to repression of Bv8 and reduced levels of pro-angiogenic bone marrow-derived CD11b+ GR1+ myeloid cells in circulation and in the tumor microenvironment. These data may provide molecular instructions to address the unmet medical need for blocking tumor progression in an early stage and developing novel early tumor dormancy biomarkers.

## Results

Four different human tumor dormancy *in-vivo* models have been established and phenotypically characterized in our laboratory. These include breast carcinoma (MDA-MB-436), glioblastoma multiforme (GBM, T98G), osteosarcoma (KHOS-24OS) and liposarcoma (SW872) human tumor cell lines. During the dormancy period of these tumors, which is characterized by impaired tumor angiogenesis, tumor cell proliferation is balanced by a high rate of apoptosis (Figure 1A). From each tumor type, we isolated and established cell lines that when injected to mice can form either dormant or fast-growing tumors [15], [16]. These cell lines were shown to maintain their characteristics when cultured *in-vitro*. According to the tumor phenotype which the cell lines generate, we hereinafter use the prefix “D” for dormant and “A” for fast-growing angiogenic tumors.

### Identification of the consensus tumor dormancy associated microRNAs

We sought to identify the consensus set of miRs that govern the transition of all four dormant tumors to the fast-growing angiogenic phenotype. Therefore, total RNA was extracted from dormant (D) and fast-growing angiogenic (A) clones of all four tumor types and subjected to high-throughput real time qRT-PCR based miR expression analysis (Figure 1B). Among the 378 miRs investigated, we found 19 miRs to be differentially regulated in the same patterns between all dormant vs. fast-growing tumors (p<0.03). Interestingly, we found a significant enrichment of upregulated miRs (84% or 16 miRs) in dormant tumors as compared to their expression levels in switched fast-growing tumors (p<0.00004 by hypergeometric distribution). These data suggest that loss of DmiR function is the key regulation pattern correlating with the ability of dormant tumors to switch to the angiogenic phenotype and grow rapidly. The 16 DmiRs identified include the *Homo sapiens* microRNAs 101, 320, 193b, 218, 151, 19a, 331, 340, 184, 186, 190, 185, 580, 588, 202 and 545. Three microRNAs (miR-520g, 657 and 92) were found to be down-regulated in dormant tumors as compared to the fast-growing tumors (Figure 1B and Information S1). For all subsequent confirmatory and functional validation studies, the top three dormancy-associated miRs – miR-580, miR-588 and miR-190– were used. These three DmiRs were selected based on their marked differential expression in dormant tumors; at least three out of four dormant tumors had an average of ∼ 12, 10 and 75-fold higher expression levels of miR-580, miR-588 and miR-190, as compared to the fast-growing tumors. The regulation of miR-580 and miR-190 was further confirmed in a second independent set of dormant vs. angiogenic tumors (Information S1).

### Regulation of DmiRs in glioma patients

To examine the regulation patterns of the identified DmiRs in primary human tumor tissue, the expression levels of miR-580, miR-588 and miR-190 in tissue specimens of glioma patients were investigated. Total RNA was isolated from three World Health Organization (WHO) grade I, four grade III and eight grade IV glioma tumors. Real time qRT-PCR analysis revealed significant correlation between miR-190 (p<0.0076) and miR-580 (p = 0.05) expression and tumor grade. As shown in Figure 1 C–D, the expression levels of miR-580 and miR-190 decreased with advanced tumor grade. Hence, the direction of DmiR regulation in primary brain tumors parallels the regulation pattern identified in the tumor dormancy models.

### Over-expression of DmiRs in fast-growing glioblastoma inhibits tumor growth

To test potential dormancy promoting functional effects of the identified DmiRs, lentiviral vectors encoding miR-580, miR-588 or miR-190 were used to reconstitute their expression in fast-growing angiogenic T98G human glioblastoma multiforme cells (A-GBM). Over-expression of DmiRs in A-GBM cells was confirmed by qRT-PCR (Information S1), and mice were injected with miR-580, miR-588, miR-190 or vector control (GFP) expressing A-GBM cells. In addition non-transfected “parental” A-GBM were used as control.

**Figure 2:**
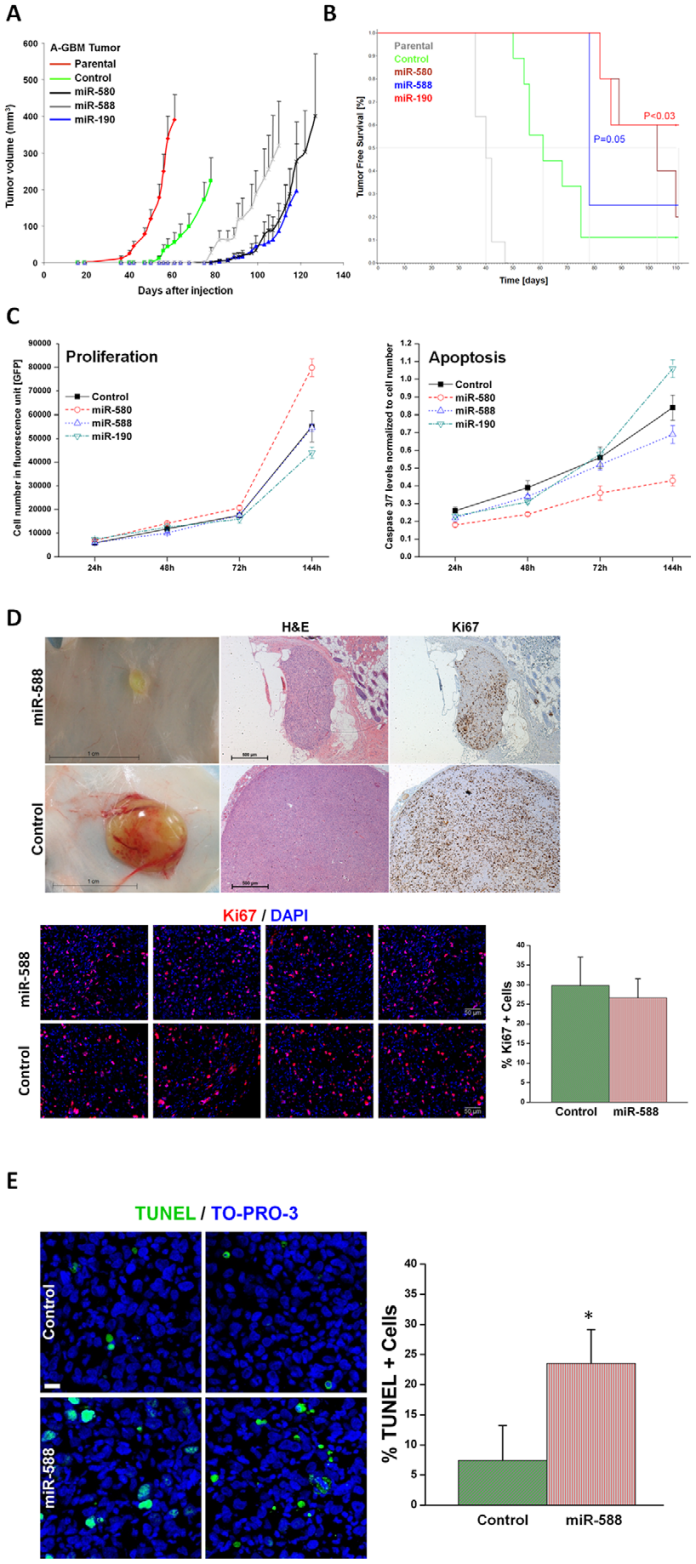
Over-expression of DmiRs reversed tumor phenotype. Tumor growth kinetics of angiogenic fast-growing glioblastoma cells (A-GBM) was monitored *in-vivo* (A). Average tumor volume of parental tumor (labeled in red line) n = 11, cells infected with GFP only control vector (n = 9), miR-580 (n = 5), miR-588 (n = 4), and miR-190 (n = 5) expressing A-GBM tumors. Kaplan-Meier analysis of tumor free survival (B). The criterion for “tumor free” was defined as mice bearing no detectable tumors by gross examination or tumors with volume smaller than 50 mm^3^. The median tumor free survival was markedly increased in miR-580 and miR-588 expressing tumors as compared to the GFP control. 60% of miR-190 expressing tumors remained dormant for the whole observation period. Statistical significance was reached for miR-190 (p<0.03) and miR-580 (p = 0.05), respectively, by log-rank test. The dormancy promoting effect of DmiRs was not attributed to impaired proliferation kinetic or enhanced apoptosis of DmiR expressing tumor cells *in-vitro* (C). In contrast, there was a trend towards higher proliferation rates and reduced apoptosis in miR-580 expressing cells (C). Proliferating tumor cells were also detected by Ki67 immunohistochemistry and representative photomicrographs are shown (D) in dormant miR-588 expressing GBMs (107days post implantation) and fast-growing GFP expressing control tumors (day 113 after implantation). *In-vivo* proliferation index was determined by counting the fraction of Ki67+ tumor cells. This analysis revealed no significant difference between the dormant miR-588 and the fast growing control (GFP) tumors (p = 0.12). Enhanced apoptosis was detected *in-vivo* in miR-588 vs. control (GFP) tumors by TUNEL staining (E). A threefold increase in the fraction of TUNEL+ apoptotic tumor cells was found in dormant miR-588 expressing- vs. control tumors (* p<0.0001).

Consistent with our previous data, the parental fast-growing A-GBM tumors were detectable between 36–42 days post injection (Figure 2A). Surprisingly, the infection of A-GBM tumor cells with negative control vector (expressing all elements as DmiR expressing vectors but lacking any microRNA sequence) modulated tumor growth kinetic and modestly delayed the time to establishment of macroscopically detectable tumors. This is assumed to be a cell type specific effect of GFP expression, as it was not evident in other tumor cell lines. However, the expression of each of the DmiRs led to a clear inhibition of tumor growth and a prolongation of the time required for tumors to become palpable. The average tumor dormancy period, i.e., the time until tumors became detectable by gross examination, was increased to ∼ 100 days by over-expression of a single DmiR (e.g., miR-580 or miR-190). Inhibition of tumor growth by expression of miR-580, miR-588 or miR-190 had also been observed in osteosaroma cell lines.

In line with these data, Kaplan-Meier analysis of the “tumor free” mice population revealed marked differences in the DmiR expressing vs. the vector control (GFP only) or parental A-GBM bearing mice. “Tumor free” mice were defined as those with no tumors detectable by gross examination or those with tumors smaller than 50 mm^3^. The median “tumor free” period, i.e., the time until tumors were detected in 50% of the monitored animals in each group, was markedly prolonged in miR-588 (∼78 days) and miR-580 (∼103 days) expressing tumors as compared to GFP-vector-control (∼61 days) or non-transfected parental A-GBM (40 days) (Figure 2B). Of note, the median survival time of the mice with miR-190 expressing tumors exceeded the observation period.

As previously described, tumor “take”, i.e., the ability to detect tumors by gross examination, correlates with the switch of tumors to the fast-growing angiogenic phenotype, whereas “no-take” indicates the existence of vital but dormant tumors. In the control groups, all parental A-GBM cell lines developed tumors (11/11, 100% tumor take rate), while ∼ 90% (8/9) of GFP expressing tumors established detectable tumors within the observation period (>140 days). In contrast, the tumor take rates were 80% (4/5) for miR-580, 75% (3/4) for miR-588 and only 40% (2/5) in miR-190 over-expressing tumors (Table 1). It is intriguing that over-expression of a single DmiR, e.g., miR-190, was sufficient to induce a complete phenotypic reversal of fast-growing A-GBM tumors towards prolonged tumor dormancy in three out of five transfected tumors. The growth dynamics of single tumors within the GFP-vector-control and miR-190 expressing tumors are presented in the Information S1.

**Table 1:** Numbers of mice with dormant “undetectable” A-GBM tumors.

microRNA	Undetectable tumors	Total number of mice	Percentage (%)
Fast growing ‘parental’	0	11	0
GFP (Control)	1	9	11
miR-580	1	4	25
miR-588	1	5	20
miR-190	3	5	60

Number of mice bearing undetectable tumors or tumors with volume smaller than 50 mm^3^ at the end point of experiments: day 64 for parental fast-growing A-GMB tumors, day 107 for control GFP expressing tumors, day 113 for miR-588 tumors, day 118 for miR-190 tumors, and day 127 for miR-580 tumors.

### DmiRs do not attenuate tumor cell proliferation *in-vitro* and *in-vivo*

Next, we aimed to investigate if the dormancy promoting effect of DmiRs is mediated by direct inhibition of tumor cell proliferation or enhanced apoptosis. Expression of miR-580, miR-588, miR-190 as well as the control-GFP-vector neither blocks cell proliferation nor enhances cell death even *in-vitro* at hyperconfluence state (after 144h) as determined by caspase 3/7 activity (Figure 2C). Moreover, miR-580 expressing cells showed a trend toward enhanced proliferation and reduced apoptosis as compared to GFP-control. The high proliferative capacity of DmiR expressing tumors was confirmed by Ki67 immunostaining of dormant tumors *in-vivo*. Figure 2D shows the high rate of proliferative Ki67+ cells in a representative dormant miR-588 expressing A-GBM tumor at day 113 post injection. This tumor was undetectable by gross examination and could only be detected by careful examination followed by skin-flip at the site of injection. The *in-vivo* proliferation index for miR-588 expressing vs. control (GFP) A-GBM tumors was assessed by counting the fraction of Ki67+ tumor cells in 15 (miR-588) and 13 (control) representative high power fields (20x). This analysis revealed no significant difference in proliferation between the two groups (p = 0.12) with a trend toward more proliferation in dormant miR-588 expressing tumors. These data suggest that the tumor dormancy promoting effect of DmiRs could not be attributed to the direct blockage of tumor cell proliferation. The *in-vivo* apoptosis index for miR-588 expressing vs. control (GFP) A-GBM tumors was assessed by counting the fraction of TUNEL+ tumor cells in 16 (miR-588) and 18 (control) representative high power fields (20x). In line with our previous reports in these dormancy models, the fraction of TUNEL positive apoptotic tumor cells was threefold increased *in-vivo* in dormant miR-588 expressing tumors vs. GFP-control tumors (p<0.0001, Figure 2E).

### Complete phenotypic reversal after miR-190 expression in fast-growing osteosarcoma

**Figure 3:**
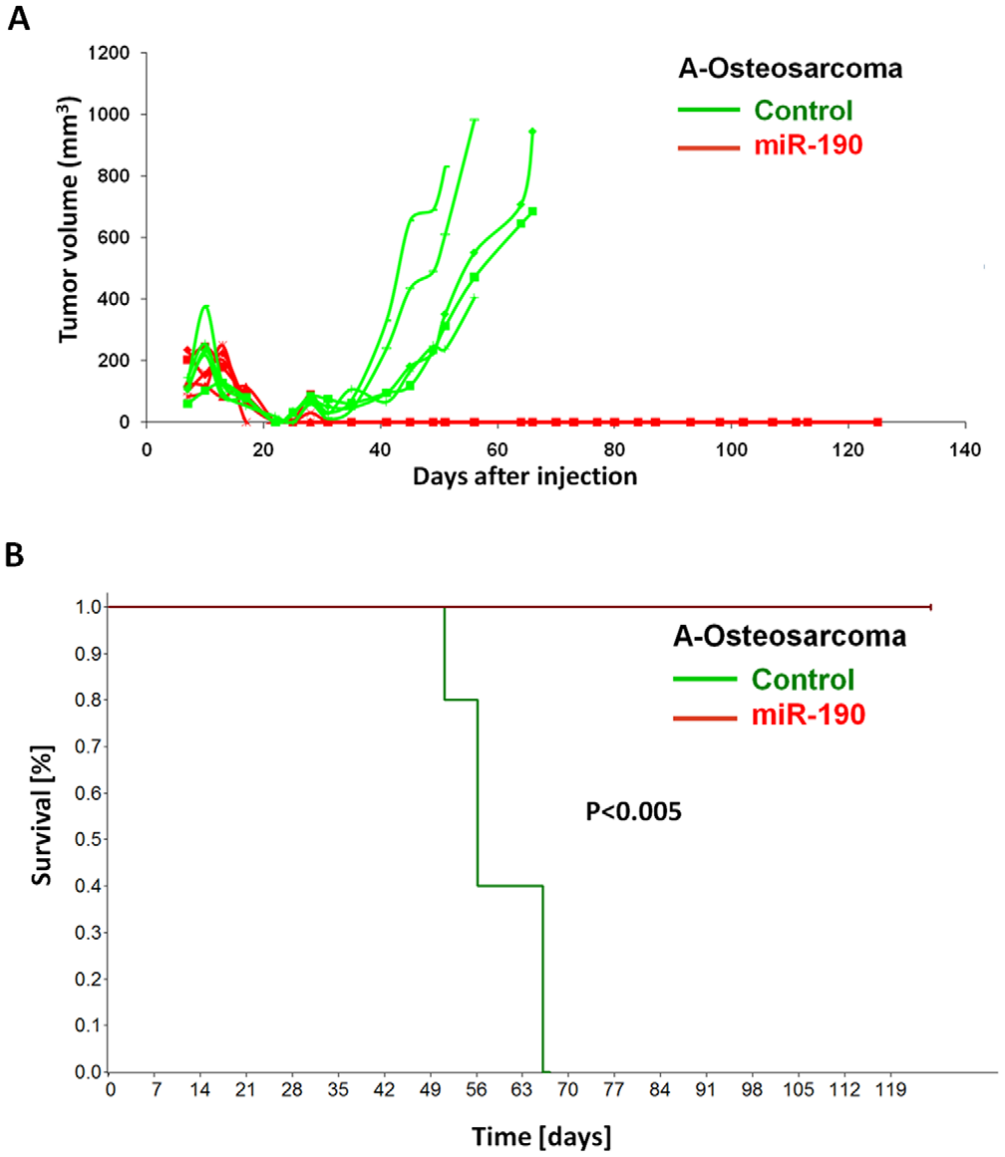
MiR-190 expression in osteosarcoma model. Tumor growth kinetic of vector control (GFP; n = 5) vs. miR-190 (n = 5) expressing angiogenic fast-growing “A-Osteosarcoma” (A). MiR-190 expression led to complete (i.e., 100% inhibition, 5/5) phenotypic reversal of fast-growing osteosarcoma resulting in significant increase of overall survival (p<0.005 by log-rank test) (B). Death event: mouse sacrificed based on large tumor size.

MicroRNA-190 was the most effective dormancy promoting DmiR in our GBM model. The tumor model with the most significant differential regulation of this DmiR was the KHOS-24OS osteosarcoma. We detected more than 210-fold higher expression levels of miR-190 in dormant vs. fast-growing osteosarcoma tumor cells (Table S1). Over-expression of miR-190 in fast-growing osteosarcoma cells led to complete inhibition of osteosarcoma growth (Figure 3). During the observation period of >120 days, no tumor was detected by gross examination in miR-190 expressing osteoscaroma (0/5), whereas the vector control GFP osteosarcoma exhibited 100% tumor take (5/5). In line with this observation, overall survival was significantly prolonged in miR-190 vs. control group (p<0.005). Together, these data support the proposed potent dormancy promoting effect of miR-190.

### Identification of the consensus transcriptional targets of DmiRs

**Figure 4:**
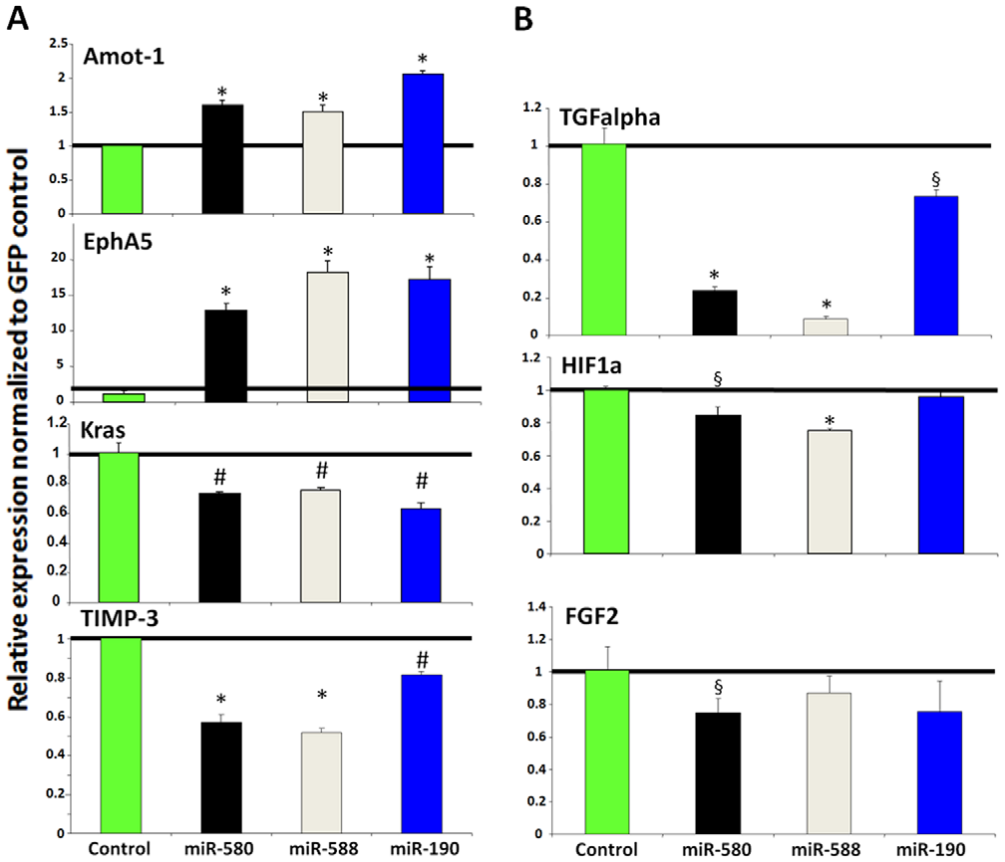
Differential expression of angiogenesis and dormancy related genes in DmiR expressing tumors. qRT-PCR analysis of gene expression in glioblastoma cells. Expression levels of genes in miR-580, miR-588 and miR-190 over-expressing A-GBM tumors were normalized to GFP-vector control. DmiR expression induced a transcriptional switch towards antiangiogenesis by down-regulation of multiple angiogenesis related genes (e.g., bFGF (FGF2), TIMP-3, HIF1A and TGFalpha). In contrast antiangiogenic and dormancy promoting pathways were upregulated after DmiR-expression (e.g., Amot-1 or EphA5). These experiments were performed in triplicates and repeated at least twice to confirm similar pattern of differential expression. Significant differential expression as compared to GFP-control is indicated with p<0.001, # p≤0.01 and § p<0.05.

**Figure 5:**
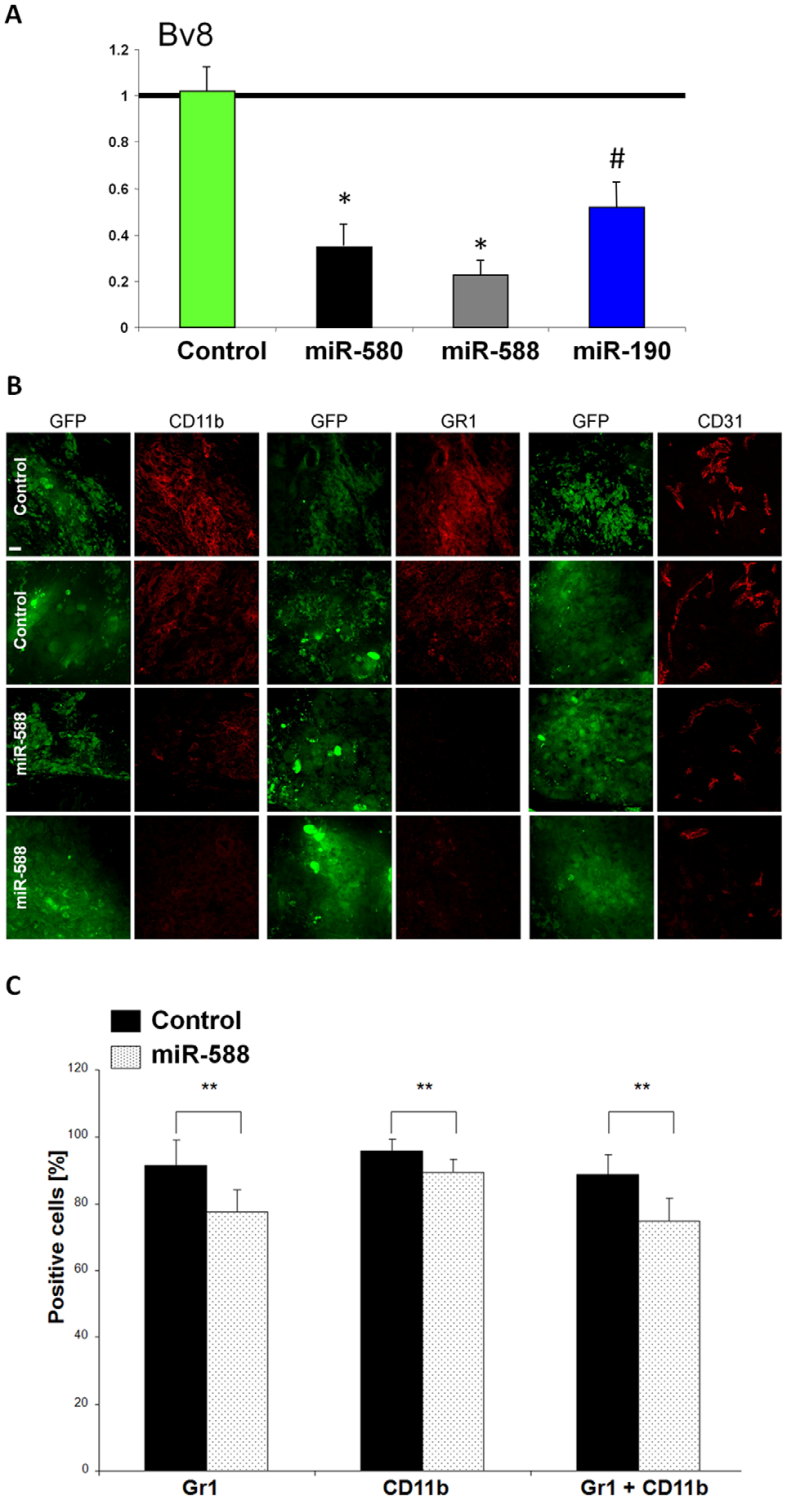
Reduced recruitment of myeloid BMDCs in DmiR expressing tumors. RT-PCR analysis of Bv8 level in DmiR over-expressing cells (A). All three DmiRs potently inhibit the expression of Bv8. Evaluation of Gr1 and CD11b positive bone marrow-derived myeloid cells in DmiR vs. control (GFP) expressing A-GBM tumors (B). MiR-588-GFP or vector-control- GFP expressing A-GBM tumors were stained for Gr1/CD11b myeloid cell- or CD31 vascular endothelial marker. Images of the same plain with detectors for GFP or antibody staining are shown. Scale bar represent 20 µm. Tumors were collected 73 days following injection to mice. Images were taken as representatives of at least three different tumors per group. Impaired mobilization of Gr1 and CD11b positive myeloid cells in circulation of miR-588 tumor bearing mice (C). Percentage of Gr1 or CD11b expressing cells among CD45 positive cells in circulation of mice bearing fast-growing glioblastoma expressing either GFP (black bars) or miR-588 (dotted bars) was determined using FACS analysis. Error bars represent SEM, ** p<0.02.

To identify the consensus target genes of DmiRs, the expression of a panel of angiogenesis- and dormancy related genes was profiled by qRT-PCR. These genes were in part selected based on the consensus transcriptional signature that we recently reported to participate in the switch of dormant tumors to the fast-growing angiogenic phenotype [17]. We found that antiangiogenic and dormancy promoting genes, Angiomotin (AMOT-1) and Eph receptor A5 (EphA5), were both upregulated in all DmiR expressing A-GBM tumors as compared to the GFP-vector-control A-GBM cells (Figure 4). In contrast, genes involved in pro-angiogenic signaling, including tissue inhibitor of metalloproteinases 3 (TIMP-3), hypoxia-induced factor 1 alpha (HIF-1-alpha), basic fibroblast growth factor (bFGF, FGF2), and the K-ras tumor oncogene, were consistently downregulated in DmiR expressing A-GBM. We found transforming growth factor alpha (TGF-α) as a common target of all three DmiR expressing A-GBM. Importantly, we found Bv8 also known as prokineticin 2 (Prok2) to be markedly downregulated in all three DmiR expressing A-GBM (Figure 5A). Bv8 has been recently discovered to play a key role in myeloid cell-dependent tumor angiogenesis and in the angiogenic switch [20], [21]. Therefore, concerted downregulation of Bv8 by all three DmiRs led us to investigate the contribution of bone marrow-derived cells (BMDCs) in the reversal of the angiogenic phenotype observed in DmiR expressing A-GBM tumors.

### DmiR induced Bv8 down-regulation correlates with decreased recruitment of myeloid cells

We sought to investigate the role of Bv8 down-regulation as a consensus target of DmiR 1-3 expression in A-GBM tumors by analyzing the mobilization and tumor recruitment of bone marrow-derived CD11b+ and Gr1+ myeloid cells. Size-matched GFP-vector-control and miR-588 expressing A-GBM tumors were harvested at day 35 post injection. Tumors were stained for Gr1+, CD11b+ (myeloid) or CD31+ (endothelial) cells (Figure 5B). CD11b+ and Gr1+ myeloid cells were highly abundant in control A-GBM tumors, whereas their level was markedly reduced in miR-588 expressing tumors. We found that miR-588 expression and down-regulation of Bv8 preferentially impaired the recruitment of the Gr1+ myeloid cell population. Gr1+ myeloid cells were almost absent in miR-588 expressing tumors (Figure 5B). The reduced recruitment of Gr1+ myeloid cells also correlated with impaired angiogenesis in miR-588 expressing tumors as detected by CD31 staining of the tumor microvascular endothelium.

To detect potential effects of DmiRs on bone marrow mobilization, we examined the circulating levels of CD11b+ Gr1+ myeloid cells. In line with the reduced recruitment of these cells detected in the tumor microenvironment, we found decreased levels of Gr1+/CD11b+ cells in circulation in miR-588 expressing tumors as compared to the levels in GFP-vector-control A-GBM (Figure 5C). Downregulation of Bv8 by DmiRs was clearly correlated with reduced mobilization and tumor recruitment of bone marrow derived CD11b+ Gr1+ myeloid cells, as well as with impaired angiogenesis.

## Discussion

We report here the discovery of a consensus set of 19 micro RNAs that are significantly regulated during the transition of four different human dormant tumors to exponential growth. Sixteen miR were found with high expression levels in dormant tumors. Downregulation of these dormancy associated miRs (DmiRs) correlated with the switch of dormant tumors to the fast-growing angiogenic phenotype. The top three DmiRs were selected based on consistent high expression levels in dormant tumors. In support of the data from the experimental dormancy models, miR-580 and miR-190 expression was shown to correlate with disease stage in human glioma specimens. Their expression was gradually decreased with advanced tumor grade suggesting a tumor suppressive function for the identified dormancy associated miRs.

Based on the preponderance of upregulated miRs in dormant vs. fast-growing tumors (16 out of 19 miRs found), we hypothesized that loss of DmiRs constitutes the prevailing regulation pattern correlating with the switch of dormant tumors to a stage of exponential growth. To prove this hypothesis, we reconstituted the expression of miR-580, miR-588 or miR-190 in fast-growing A-GBM tumors. Moreover, we tested the expression of miR-190 in a fast-growing A-Osteosarcoma model. To our knowledge, this is the first report demonstrating the phenotypic reversal of fast-growing human tumors back to the dormancy phase by over-expression of a single miR. It is intriguing that over-expression of a single miR, in particular miR-190, resulted in significant delay of time to tumor establishment in 40% and in sustained dormancy in 60% of GBM tumors, i.e., no detectable tumors were found by gross examination up to 140 days post injection. Moreover, miR-190 expression led to complete phenotypic reversal in 100% of fast-growing A-Osteosarcoma. These dramatic effects might be attributed to the unique ability of single miRs to regulate expression levels of a large number of genes [22], [23]. Unfortunately, the knowledge of the function and potential gene targets of miR-580, miR-588 and miR-190 is very limited and their contribution to cancer progression is unknown. Therefore, identification of potential miR targets in this relatively new field of gene regulation relies mostly on bioinformatics prediction.

To dissect the molecular mechanism underlying the phenotypic reversal of DmiR expressing A-GBM tumors, the expression level of a selected set of genes was profiled. These genes were either involved in the angiogenesis process or were recently shown to participate in the switch of dormant tumors to the angiogenic phenotype [17]. We further aimed to identify common targets of all three DmiRs. Among the consensus miR-580, miR-588 and miR-190 upregulated transcripts, we found two important dormancy associated genes, i.e., EphA5 and Angiomotin. Angiostatin, a key endogenous angiogenesis inhibitor and dormancy promoting factor [24]–[26], was shown to be preferentially enriched in angiomotin positive dormant tumors [17]. Likewise, EphA5 was previously shown to be expressed in dormant tumors, downregulated once they switch to the angiogenic phenotype, and to gradually decrease with advanced tumor stage in tumor specimens of glioma patients [17]. Moreover, detection of circulating EphA5 protein levels correlated with tumor dormancy phase. The switch of dormant GBM tumors to fast-growth resulted in a significant decrease of plasma EphA5 levels suggesting this protein as a novel dormancy biomarker [17]. Therefore, our data reinforce the importance of these two proteins as common targets of all three tested DmiRs and suggest their involvement in regulation of the dormancy process.

On the other hand, we found key angiogenic proteins (e.g., bFGF, TGFalpha or HIF1-alpha) as common downregulated targets of the functionally investigated DmiRs. The miR regulation of HIF-1 is an interesting finding because this key hypoxia sensor and upstream regulator of a number of angiogenic factors is believed to be predominantly regulated by post-translational mechanism [27]. However, a growing body of data indicates downregulation of HIF1 by endogenous or pharmacologic antiangiogenesis [28]–[30]. Inhibition of hypoxia-induced angiogenesis via DmiRs could provide a novel strategy to control the expansion of tumor mass beyond the oxygen diffusion limit (100–200µm). Our data thus support the hypothesis that tumor dormancy may result from the inability of tumor cells to induce or sustain tumor angiogenesis [1], [12], [31]. MiR-580, miR-588 and miR-190 also downregulated TIMP-3, an endogenous inhibitor of metalloproteinases, that was recently found to be involved in the dormancy process. In line with our data, TIMP-3 expression levels were shown to increase with the progression of dormant tumors towards fast-growth [17]. These data suggest differential expression of dormancy- and angiogenesis related genes as relevant targets of DmiR-induced tumor dormancy.

Congruent with these observations, we found that *in-vitro* and *in-vivo* tumor cell proliferation was not attenuated by over-expression of DmiRs. Hence, we postulated that tumor-microenvironment communication could be the principal target of the identified DmiRs and the perturbation of tumor niche might contribute to the “escape” of tumors from dormancy. In this context, identification of Bv8 as a downstream downregulated target of all three DmiRs was an important finding. Of note, the expression of the positive regulator of Bv8, the granulocyte colony-stimulating factor (G-CSF), was undetectable in all GBM cells tested (data not shown). Hence, our data suggest that that miR-580, miR-588 and miR-190 downregulate Bv8 in a GCSF independent manner. Bv8 was recently reported as a key mediator of bone marrow-derived myeloid cell-dependent tumor angiogenesis [20], [32]. Therapeutically, anti-Bv8 treatment was shown to be most efficacious when initiated in the early stages of tumor development. These data led us to investigate the contribution of bone marrow-derived cells (BMDCs) in the reversal of the angiogenic phenotype observed in DmiR expressing A-GBM tumors. We found that expression of DmiRs resulted in marked decrease of CD11b+ GR1+ myeloid BMDCs in the tumor microenvironment. Accordingly, the bone marrow mobilization of these cells was attenuated in DmiR expressing tumor bearing mice resulting in reduced circulating CD11b+ GR1+ myeloid cell levels. The bone marrow-derived CD11b+ myeloid cells constitute a relatively heterogenous cell population. Our data indicate that DmiRs might elicit their dormancy promoting effect via preferential blockage of the Gr1+ positive subpopulation. Together, our data suggest that the dormancy promoting effect of DmiRs could, at least in part, be attributed to their ability to block tumor recruitment of BMDCs via downregulation of Bv8.

Consistent with our findings, a growing body of data suggests a critical contribution of various BMDC populations in tumor formation and angiogenesis [33]. It has been recently reported that tumor recruitment of immature CD11c + dendritic cells correlates with enhanced angiogenesis and the switch of dormant breast tumors to the fast-growing phenotype [34]. Further, recruitment of Lin-/Sca1+/cKit+ bone marrow derived cells was associated with “instigation” of otherwise indolent tumors [35]. Together with our data, it is conceivable that additional bone marrow derived cell populations will be identified to participate in the exit of tumors from tumor dormancy.

Using a phenotypic assisted miR profiling strategy, we could successfully identify a set of miRs differentially regulated during the switch of dormant tumors to exponential growth. The phenotypic reversal of fast-growing tumors by over-expression of three candidate DmiRs encourages in-depth investigation of additional dormancy associated miRs discovered in this study. Our data indicate that development of a tumor “permissive niche” is a critical step for the exit of dormant tumors to the fast-growing angiogenic phenotype. Generation of a non-permissive tumor niche via alteration of tumor transcriptome and modulation of tumor stroma by miRs may constitute a promising strategy to prevent cancer or to reverse malignant tumors into an asymptomatic dormant stage.

## Materials and Methods

### Ethics Statement

Studies performed on human specimens were approved by the local ethics committee of the Charité – Universitaetsmedizin Berlin, Berlin, Germany, and written informed consent was obtained from all patients. All animal work was approved and performed in accordance with the standards of the St. Elizabeth's Medical Center, Boston, MA, USA, Institutional Animal Care and Use Committee (IACUC).

### Cell lines, tissue culture and surgical specimens

Human breast adenocarcinoma (MDA-MB-436), osteosarcoma (KHOS-24OS), glioblastoma (T98G), and liposarcoma (SW872) cell lines were obtained from the American Type Culture Collection (ATCC, Manassas, VA). Dormant and angiogenic fast-growing populations were generated and maintained as previously described [15]–[17]. Cell proliferation assay was performed using cellular GFP-expression or CyQUANT NF Cell Proliferation Assay Kit (Molecular Probes, Invitrogen, CA) according to manufacturer's instructions. Apoptosis was detected by Caspase3/7 activity using Caspase - Glo 3/7 Assay (Promega, Mannheim, Germany) according to manufacturer's instructions. Glioma specimens were obtained from surgical tumor resections. The diagnosis was made according to the criteria of the WHO. Samples were immediately snap frozen in isopentane, precooled over liquid nitrogen, and stored at −80°C until further processing.

### Animals and tumor cell inoculation

Tumor cells were injected subcutaneously into the lower right quadrant of the flank of each mouse as previously described [16]. Male SCID mice aged 6–8 weeks (Charles River Laboratories, MA) were cared for in accordance with the standards of the St. Elizabeth's Medical Center Institutional Animal Care and Use Committee (IACUC). Tumor volume was calculated using the standard formula: length×width^2^×0.52.

### Total-RNA isolation and quality control

Total-RNA, including miRs, was isolated using TRIzol (Invitrogen) according to the manufacturer's protocol. RNA integrity and concentration was determined using RNA 6000 Nano Lab on Chip kits and Agilent 2100 Bioanalyzer (Agilent, CA).

### MicroRNA expression analysis

Real-time quantitative miR expression analysis was performed using TaqMan assays (Applied Biosystems, ABI, CA). First, miRs were converted into cDNA via sequence specific reverse transcription (RT) reactions using Multiplex RT MicroRNA Kit (ABI). C-DNA was then loaded into the low density (384 well) micro fluidic cards that were pre-loaded with TaqMan probes (Human MicroRNA Panel v1.0, Applied Biosystems) and microRNA expression level were analyzed using a 7900HT Fast Real-Time PCR System (ABI) according to the manufacturer's protocol.

### Stable over-expression of miRs in tumor cells

Target microRNAs were introduced into tumor cells via the Lenit-miR system (System Biosciences, CA). Briefly, pre-microRNA constructs were cloned into a lentiviral vector and used together with pPACKH1 Lentivector Packaging Kit and 293TN Packaging Cell Line (System Biosciences, CA) to generate virus particles. Human tumor cells were infected with virus particles according to manufacturer's recommendations. Stable expression of DmiRs in A-GBM cells was monitored via green fluorescence protein (GFP) expression and GFP positive cells were selected via fluorescence-activated cell sorting (FACS) prior to *in-vitro* and *in-vivo* experiments. GFP expression was monitored throughout tumor growth under tissue culture conditions and also validated *in-vivo* at the time point of tumor harvest.

### Antibodies and FACS analysis

Antibodies were purchased from BD Pharmingen (Franklin Lakes, NJ) and included rat anti-mouse CD11b antibody (clone m1/70), purified rat anti-mouse Ly-6G /Ly-6C (anti Gr1 antibody, clone RB6-8C5), anti-mouse CD45 (LCA, Ly-5, BO-F11) and rat anti-mouse CD31 antibody. FACS analysis was performed using peripheral blood collected by cardiac puncture of tumor bearing mice, 0.9 ml blood was collected into a 0.1 citrate buffer tube, and incubated with Lysis buffer (RBC Lysis buffer, BioLegend). Following centrifugation, supernatant was collected and resuspended in FACS wash buffer (PBS, 0.1% NaN3, 1% fetal bovine serum). 1 µL of each primary antibody was added to 50 µL of blood and incubated for 30 min on ice. Gr1+ and CD11b+ cell fractions of CD45+ cells were analyzed.

### Immunofluorescence staining and microscopy

Tumor tissues were stored in OCT (Tissue Tek, Sakura Finetek Europe, The Netherlands). For CD31 antibody, the tissue sections were rinsed once in 1x PBS and were fixed by first placing them in 80% methanol at −20°C for 10min and then in 4% PFA for 5min at room temperature (RT). For all other antibodies, tissue was rinsed once in 1x PBS and fixed in 4% PFA for 5min at RT. First antibodies were incubated at 4°C over night. Alexa Fluor 555 secondary antibody (Molecular Probes, Carlsbad, CA) was used at 1∶300 dilution in 10% goat serum for 1h in dark. For Ki67 studies, rabbit anti Ki67 (ab833, abcam, Cambridge, UK) and goat anti-rabbit Alexa Fluor 594 (Invitrogen, A31632) were used. Nuclear staining was done using DAPI or To-Pro-3 (Molecular Probes). TUNEL staining was performed using Click-it TUNEL Alexa Fluor 594 Imagin Assay (Invitrogen) according to manufacturer instructions. Microscopy was performed using a confocal laser scanning system (Zeiss LSM 510 Meta, Carl Zeiss, Jena, Germany) and Zeiss software.

### Statistical methods

Statistical analysis of real-time quantitative miR data, Kaplan-Meier (KM) graphs and pairwise log-rank tests were computed with Statistical Utility for Microarray and Omics data (SUMO) software package (HUhttp://www.oncoexpress.org/software/sumoUH). The time point at which the tumor was first detectable by gross examination was used to build the KM estimator. Those animals which did not undergo this switch within the experimental period, where added as “censored” at the last time point of data acquisition (day 181). Data are presented as mean ± SEM unless otherwise noted. Statistical significance was assessed using Student's *t* test unless otherwise noted. *P*<0.05 was considered statistically significant. All statistical tests were two-tailed.

## Supporting Information

Information S1 Supporting Information **Figure S1.** Confirmation of miR-580 and miR-190 regulation in an independent tumor set. Expression levels of tumor dormancy associated microRNAs were analyzed using real-time qRT-PCR and compared between dormant and fast-growing tumor cells of each cancer type. **Figure S2.** Over-expression of miR-580, miR-588 and miR-190 in fast-growing angiogenic glioblastoma. Using qRT-PCR the efficacy of DmiR transfection was confirmed by analyzing their expression levels in miR-580, miR-588 or miR-190 vs. GFP-vector-control infected fast-growing glioblastoma multiforme tumors (A-GBM). **Figure S3.** Tumor growth was compared between GFP and miR-190 over-expressing clones of fast growing glioblastoma (A-GBM). Each line represents one tumor. Green lines represent GFP expressing tumors while blue lines represent miR-190 over-expressing clones. N =  5 per group.
(PDF)

Table S1 **Differential expression of all miRs profiled in dormant vs. fast-growing tumors.**
(DOC)

Methods S1 **Real time quantitative reverse transcription-PCR (qRT-PCR) analysis of single microRNA targets.** All assays were performed according to Applied Biosystems TaqMan MicroRNA Assays Protocol recommendation. Briefly, 10ng (5ul) of total RNA was taken per 15-ul RT reaction. Reverse transcription was performed using MultiScribe™ Reverse Transcriptase (Applied Biosystems) and TaqMan MicroRNA Reverse Transcription Kit (Applied Biosystems). RT reaction products were diluted 1:10 and 2 microliter were taken for PCR amplification reaction together with TaqMan MicroRNA Assay specific to target microRNA and TaqMan 2x Universal PCR Master Mix (Applied Biosystems). The following TaqMan MicroRNA assays were used: has-miR-190 ( Cat #4373110,AB), has-miR-580 ( Cat #4381024,AB), has-miR-588 ( Cat #4380952,AB), has-miR-520 ( Cat #4373257,AB) and has-miR-657( Cat #4380922,AB). The has-miR-RNU6B was used as endogenous control (Cat #4373381,AB).
(DOCX)
