# Supplementary figures and images for: Consensus Micro RNAs Governing the Switch of Dormant Tumors to the Fast-Growing Angiogenic Phenotype
Source: PLoS One. 2012 Aug 31;7(8):e44001. doi: 10.1371/journal.pone.0044001 (PMC3432069; doi:10.1371/journal.pone.0044001)

Supplemental Figure 1

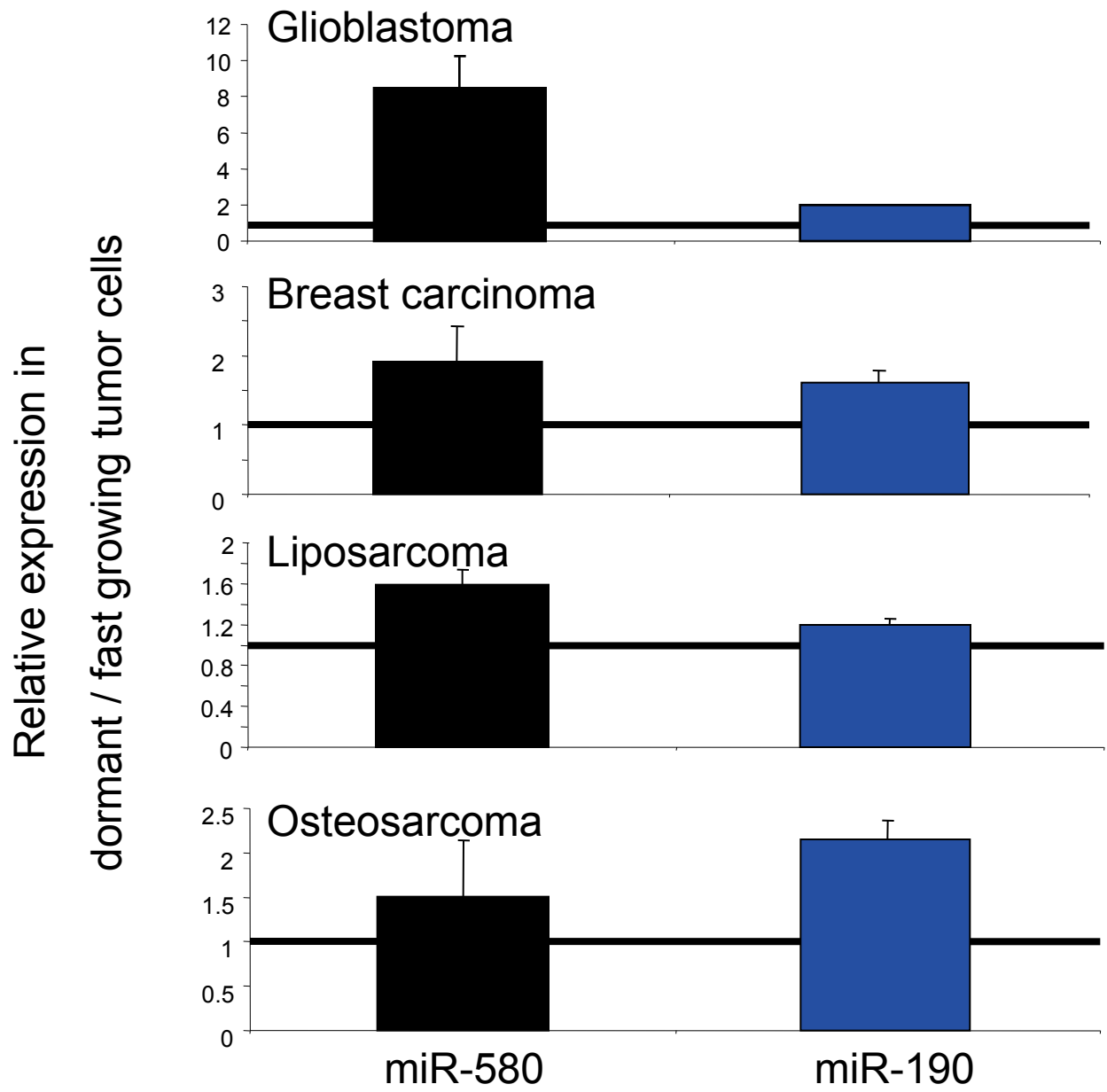

Supplemental  
Figure 2

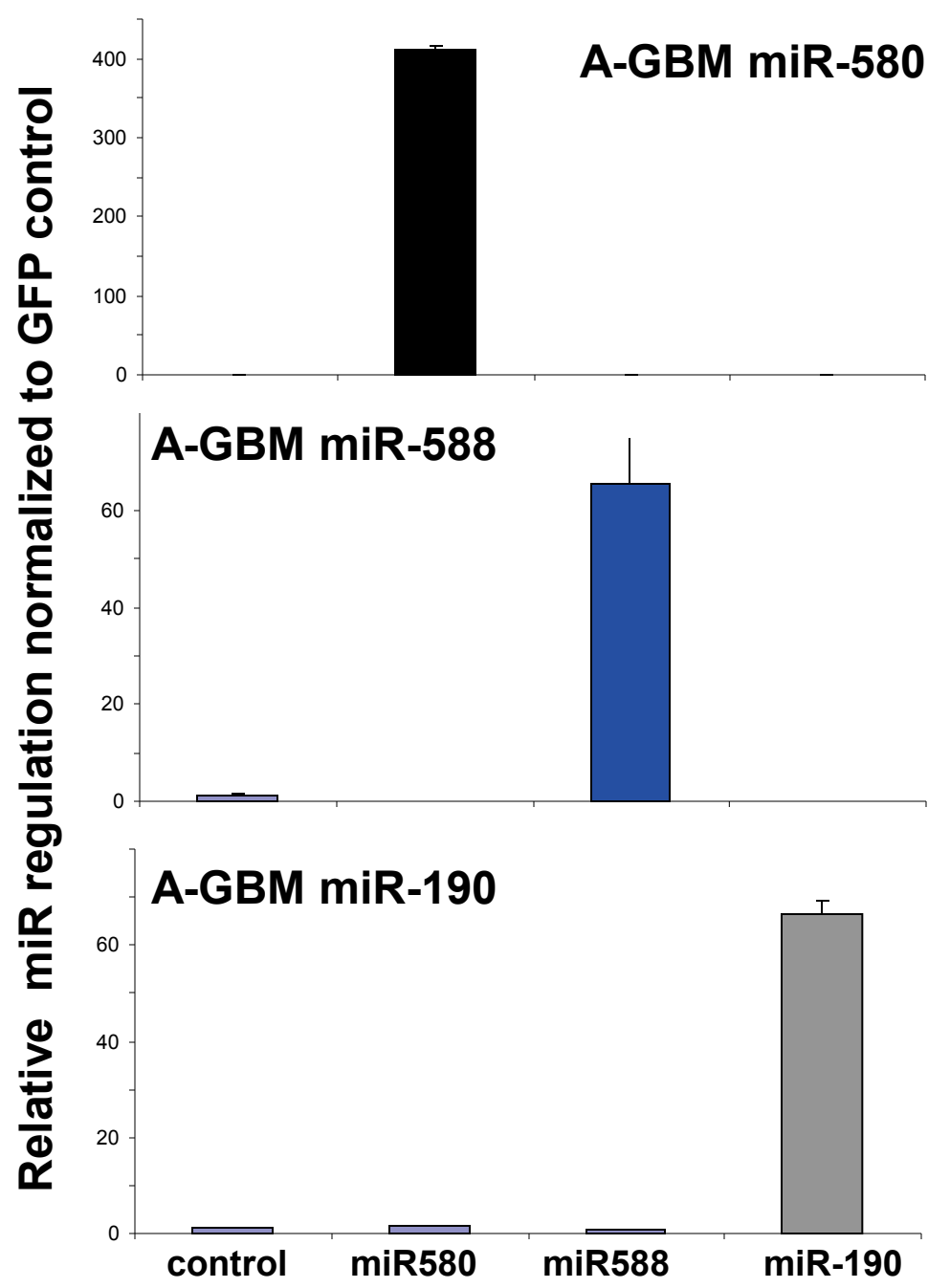

Supplemental Figure 3

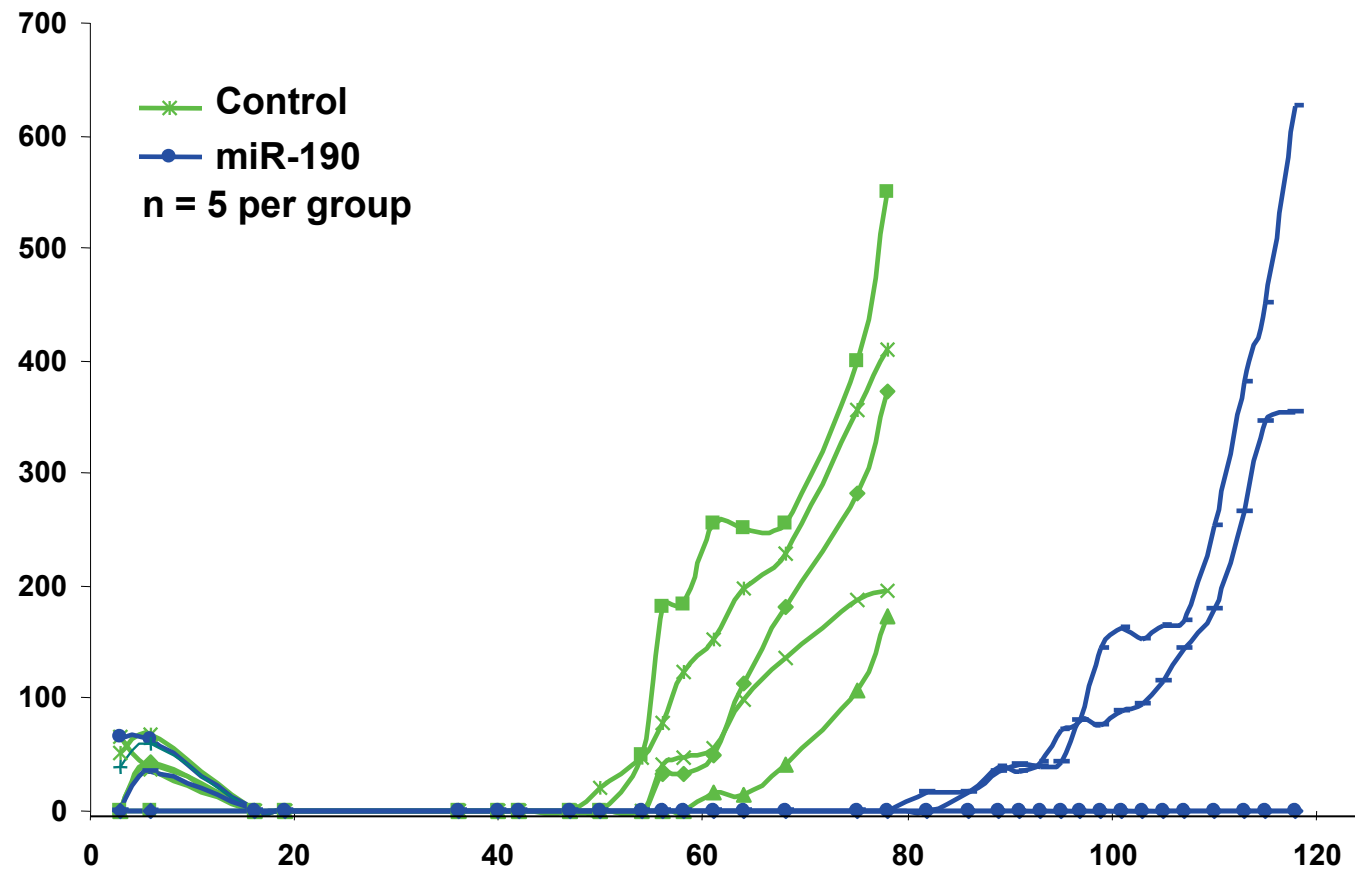

Supplement: Information S1 Supporting Information — Figure S1. Confirmation of miR-580 and miR-190 regulation in an independent tumor set. Expression levels of tumor dormancy associated microRNAs were analyzed using real-time qRT-PCR and compared between dormant and fast-growing tumor cells of each cancer type. Figure S2. Over-expression of miR-580, miR-588 and miR-190 in fast-growing angiogenic glioblastoma. Using qRT-PCR the efficacy of DmiR transfection was confirmed by analyzing their expression levels in miR-580, miR-588 or miR-190 vs. GFP-vector-control infected fast-growing glioblastoma multiforme tumors (A-GBM). Figure S3. Tumor growth was compared between GFP and miR-190 over-expressing clones of fast growing glioblastoma (A-GBM). Each line represents one tumor. Green lines represent GFP expressing tumors while blue lines represent miR-190 over-expressing clones. N = 5 per group. (PDF) [file pone.0044001.s001.pdf]
