# Supplementary material for: Consensus Micro RNAs Governing the Switch of Dormant Tumors to the Fast-Growing Angiogenic Phenotype
Source: PLoS One. 2012 Aug 31;7(8):e44001. doi: 10.1371/journal.pone.0044001 (PMC3432069; doi:10.1371/journal.pone.0044001)
Supplement: Table S1 — Differential expression of all miRs profiled in dormant vs. fast-growing tumors. (DOC) [file pone.0044001.s002.doc]

**Supplemental table 1: Fold differences of miR expression**

| **MicroRNA** | **Liposarcoma** | **Breast Ca.** | **Glioblastoma** | **Osteosarcoma** |
| --- | --- | --- | --- | --- |
| 193b | 1.3 | 1.4 | 1.1 | 1.8 |
| 19a | 1.8 | 1.2 | 1.5 | 1.7 |
| 101 | 1.6 | 1.3 | 1.4 | 2.5 |
| 151 | 1.4 | 1.1 | 1.2 | 1.1 |
| 184 | 2.1 | 1.2 | 2.8 | 3.9 |
| 185 | 4.3 | 2.6 | 1.1 | 2.3 |
| 186 | 1.9 | 1 | 2.4 | 2.4 |
| **190** | **73.4** | **2.2** | **14.5** | **210.7** |
| 202 | 1.2 | 5.9 | 5.8 | 16.9 |
| 218 | 3.3 | 1.4 | 1.5 | 2.8 |
| 320 | 1.5 | 1.2 | 1.3 | 1.8 |
| 331 | 1.6 | 1.1 | 1.6 | 1.4 |
| 340 | 2.2 | 1.7 | 2.7 | 3.2 |
| 545 | 1.9 | 2.1 | 12.6 | 4.2 |
| **580** | **8.8** | **11.7** | **N.A.** | **16.8** |
| **588** | **9.8** | **12.9** | **N.A.** | **8.5** |
| 657 | 30.5 | 24 | N.A. | 16.1 |
| 92 | 1.2 | 1.4 | 1.5 | 1.5 |
| 520g | 15.2 | 23.4 | N.A. | 16.4 |

Fold difference of miR expression between dormant- and fast-growing tumors cells obtained by real-time quantitative PCR. The most significantly regulated microRNAs (p<0.03) are presented. 19 microRNAs were up-regulated in dormant tumors (ratios indicate expression levels in dormant vs. fast growing tumors). MicroRNAs 657, 92 and 520g, below the dashed line, were down-regulated in dormant tumors i.e., their ratios indicate miR expression level in fast-growing as compared to dormant tumors. N.A. indicates microRNA expression too low to be accurately determined by real-time PCR.
