# Supplementary material for: Consensus Micro RNAs Governing the Switch of Dormant Tumors to the Fast-Growing Angiogenic Phenotype
Source: PLoS One. 2012 Aug 31;7(8):e44001. doi: 10.1371/journal.pone.0044001 (PMC3432069; doi:10.1371/journal.pone.0044001)
Supplement: Methods S1 — Real time quantitative reverse transcription-PCR (qRT-PCR) analysis of single microRNA targets. All assays were performed according to Applied Biosystems TaqMan MicroRNA Assays Protocol recommendation. Briefly, 10ng (5ul) of total RNA was taken per 15-ul RT reaction. Reverse transcription was performed using MultiScribe™ Reverse Transcriptase (Applied Biosystems) and TaqMan MicroRNA Reverse Transcription Kit (Applied Biosystems). RT reaction products were diluted 1:10 and 2 microliter were taken for PCR amplification reaction together with TaqMan MicroRNA Assay specific to target microRNA and TaqMan 2x Universal PCR Master Mix (Applied Biosystems). The following TaqMan MicroRNA assays were used: has-miR-190 ( Cat #4373110,AB), has-miR-580 ( Cat #4381024,AB), has-miR-588 ( Cat #4380952,AB), has-miR-520 ( Cat #4373257,AB) and has-miR-657( Cat #4380922,AB). The has-miR-RNU6B was used as endogenous control (Cat #4373381,AB). (DOCX) [file pone.0044001.s003.docx]

**Real time quantitative reverse transcription-PCR (qRT-PCR) analysis of single microRNA targets.**

All assays were performed according to Applied Biosystems TaqMan MicroRNA Assays Protocol recommendation. Briefly, 10ng (5ul) of total RNA was taken per 15-ul RT reaction. Reverse transcription was performed using MultiScribe™ Reverse Transcriptase (Applied Biosystems) and TaqMan MicroRNA Reverse Transcription Kit (Applied Biosystems). RT reaction products were diluted 1:10 and 2 microliter were taken for PCR amplification reaction together with TaqMan MicroRNA Assay specific to target microRNA and TaqMan 2x Universal PCR Master Mix (Applied Biosystems). The following TaqMan MicroRNA assays were used: has-miR-190 ( Cat #4373110,AB), has-miR-580 ( Cat #4381024,AB), has-miR-588 ( Cat #4380952,AB), has-miR-520 ( Cat #4373257,AB) and has-miR-657( Cat #4380922,AB). The has-miR-RNU6B was used as endogenous control (Cat #4373381,AB).
